# Diagnostic value of respiratory virus detection in symptomatic children using real-time PCR

**DOI:** 10.1186/1743-422X-9-276

**Published:** 2012-11-19

**Authors:** Elisabeth G Huijskens, Renée C Biesmans, Anton G Buiting, Charles C Obihara, John W Rossen

**Affiliations:** 1 Laboratory of Medical Microbiology and Immunology, St Elisabeth Hospital, Tilburg, The Netherlands; 2 Department of Pediatrics, St. Elisabeth Hospital, Tilburg, The Netherlands; 3 Department of Medical Microbiology, University of Groningen, University Medical Center Groningen, P.O. 30.001, Groningen, RB, 9700, The Netherlands

**Keywords:** Respiratory virus, Real-time PCR, Clinical impact, Ct value, Children

## Abstract

**Background:**

Acute respiratory tract infections are an important public health problem. Sensitive and rapid diagnostic techniques have been developed and are used in daily clinical practice. Here we evaluate the clinical relevance of detecting 20 common respiratory pathogens by molecular methods in a general pediatric clinic.

**Methods:**

Nasopharynx samples of children < 18 years of age with respiratory symptoms referred to a general pediatric clinic were tested for the presence of 19 viruses and *Mycoplasma pneumoniae*, using real-time polymerase chain reaction.

**Results:**

Of 177 patients included in this retrospective study, 73% were positive for at least one virus. Respiratory syncytial virus (36.6%) and human rhinovirus (24%) were most frequently detected. Patients in whom a respiratory virus or *Mycoplasma pneumoniae* was detected, were younger (6 versus 24 months; p < 0.001) and more often hospitalized (116 versus 34; p = 0.001) than patients in whom no respiratory pathogen was detected. Also they were more likely to present with feeding problems, dyspnea, rhinorrhea and wheezing (all p < 0.05) than patients without a respiratory pathogen.

In the majority of cases, clinicians did not change their antibiotic management after detecting a viral respiratory pathogen. No difference in mean Ct value was found between patients with one respiratory pathogen and those with >1 respiratory pathogen (30.5 versus 31.2; p = 0.573).

**Conclusion:**

Routine testing of common respiratory pathogens could lead to a better understanding of their role in disease in children with respiratory symptoms.

## Introduction

Acute respiratory tract infections (ARTIs) are a significant cause of morbidity and account for a major percentage of mortality in early childhood worldwide. Though ARTIs can be caused by bacteria and fungi, viral infections seem to be responsible for most infections. Influenza A and B virus (FLUAV, FLUBV), respiratory syncytial virus (RSV), human parainfluenza viruses (HPIVs) and adenovirus (HAdV) are well recognized respiratory pathogens that account for 35% to 87% of ARTIs in children and cannot be distinguished on the basis of clinical presentation and symptoms [1-5].

Over the past two decades, molecular diagnostic techniques for the detection of respiratory pathogens have been developed, providing rapid results with an increased sensitivity [3,4,6]. In addition, these new techniques contributed to the discovery of novel viruses such as the human metapneumovirus (HMPV) [7], SARS coronavirus [8], coronaviruses (HCoV) NL63 [9] and HKU1 [10], human bocavirus (HBoV) [11] and the recently described KI and WU polyomaviruses (KIPyV, WUPyV) [12,13]. In this study we tried to evaluate the clinical epidemiologic features of detecting 20 common respiratory pathogens, 19 viruses and *Mycoplasma pneumoniae*, in children attending a general pediatric clinic and the influence of their detection in clinical decision making.

## Materials and methods

### Study design and subjects

The study was conducted in St. Elisabeth Hospital, one of the largest non-university teaching hospitals in The Netherlands. All patients < 18 years of age who presented with signs and symptoms of upper or lower respiratory tract infection (RTI) (rhinorrhea, dyspnea, cough, apnea, wheezing, tachypnea, etc.) at the hospital emergency department, the pediatric outpatient clinic or admitted to the pediatric ward between January 2010 through December 2010 and in whom a nasopharyx sample was collected for respiratory virus and *M*. *pneumoniae* detection were included. Based on the Dutch health system, the majority of pediatric patients who visit the hospital are referred by their family doctor. Only a minority of patients who visit the emergency department are not referred.

Relevant demographic and clinical data were extracted from hospital charts using standardized case record forms. Data collected included age, gender, underlying medical conditions, use of antibiotics prior to admission, use of antibiotics at admission, signs and symptoms at presentation, findings on physical examination, oxygen requirement, rate and duration of hospitalization, intensive care unit admission, complications, bacterial cultures and definitive clinical diagnosis.

#### Ethical considerations

Both the data collection and analyses were conducted in a retrospective fashion from coded hospital medical records, for which according to the Dutch Medical Research Involving Human Subjects Act (article 1, paragraph 1, section b of the WMO) no medical ethics review was required. This was confirmed by our institutional Ethics Review Board when we sought approval. The need for subject or parental informed consent was thus waived. To guarantee the privacy of study patients we coded all tested isolates according to the requirements of the National Privacy Regulations in The Netherlands.

### Molecular detection of respiratory pathogens

All nasopharynx samples were tested with reverse transcriptase (RT) real-time PCR [(RT)-qPCR] using parallel qPCR assays for the following combinations of respiratory pathogens: 1) FLUAV/FLUBV, 2) RSV, 3) HPIV-(1–4), 4) HMPV/ human rhinovirus (HRV), 5) HCoV- HKU/NL63/OC43/229E, 6) HAdV, 7) HBoV, 8) KIPyV/WUPyV, 9) human enterovirus (HEV)/human parechovirus (HPeV), 10) *M*. *pneumoniae*. (RT)-qPCR procedures were performed as described previously [6,14,15].

All samples had been spiked before extraction with internal control viruses, Phocine Herpes Virus (PhHV; DNA virus), and Phocine Distemper Virus (PDV; RNA virus) to monitor efficient extraction and amplification. For each target a positive and negative control was used. (RT)-qPCR results were expressed in cycle threshold (Ct) values. Ct values are inversely correlated with viral load, i.e., low Ct values indicate high viral loads.

### Statistical analysis

Comparison among groups was performed using a chi-square test for categorical variables and for continuous variables a t-test or Mann–Whitney test was used with a significance level of p < 0.05. Ct values of each virus were compared between the various groups using a t-test. Whenever a given virus was not detected by (RT)-qPCR, the corresponding viral load was assigned a value of zero. Statistical analyses were conducted using PASW Statistics 18 (IBM Company, Chicago, VS).

## Results

A total of 177 nasopharynx specimens of 177 patients were collected and analyzed. Ninety-eight (55.4%) patients were males and 79 (44.6%) were females. The median age was 8 months (range 3 days to 16 years). Thirty-five (19.8%) children had an underlying medical condition. Demographic characteristics of the patients are presented in Table 1.

**Table 1:** Demographic characteristics by number of respiratory pathogens detected

	**Number of pathogens detected**					
	**0**	**1**	**2**	**3**	**4**	**Total pathogens**
Number of patients (%)	47 (26.5)	83 (46.9)	38 (21.5)	8 (4.5)	1 (0.6)	177 (100)
Median age (months, range)	24 (0–192)	4 (0–153)	7 (1–77)	14 (2–52)	9	8 (0–192)
Male (%)	20 (42.6)	46 (55.4)	27 (71.1)	5 (62.5)	0 (0)	98 (55.4)
Underlying medical conditions (%)	13 (27.7)	17 (20.5)	4 (10.5)	1 (12.5)	0 (0)	35 (19.8)
Supplemental oxygen requirement (%)	8 (17.4)	31 (37.3)	14 (36.8)	5 (62.5)	0 (0)	58 (33.0)
Intensive care unit admission (%)	1 (2.1)	3 (3.6)	1 (2.6)	0 (0)	0 (0)	5 (2.8)
Mean duration of hospitalization (days)	4.0	5.5	3.0	4.8	0	4.5
Prematurity (%)	6 (12.8)	16 (19.3)	3 (7.9)	1 (12.5)	0 (0)	26 (14.7)

### Etiology

At least one respiratory virus or *M*. *pneumoniae* was detected in 130 (73.4%) respiratory samples. Significantly more viruses were found in male (n = 78; 60.0%) than in female (n = 52; 40.0%) patients (p = 0.042). Except for FLUAV and HPIV-2, all other respiratory viruses and *M*. *pneumoniae* were detected. Most commonly detected were RSV in 64 (36.6%) and HRV in 42 (24.1%) samples. The detection rates of the other viruses were HEV in 8.5%, WUPyV in 5.6%, HPIV-1 in 5.1%, HAdV and HBoV in 4.5%, HCoV-OC43 in 4.0%, HPIV-3 in 2.8%, HPeV 2.3%, HCoV-HKU1, HMPV and *M*. *pneumoniae* in 1.7%, FLUBV in 1.1% of the samples, respectively. The detection rates for HCoV-NL63, HCoV-229E, KIPyV and HPIV-4 were <1%.

### Seasonality of respiratory virus infection

**Figure 1:**
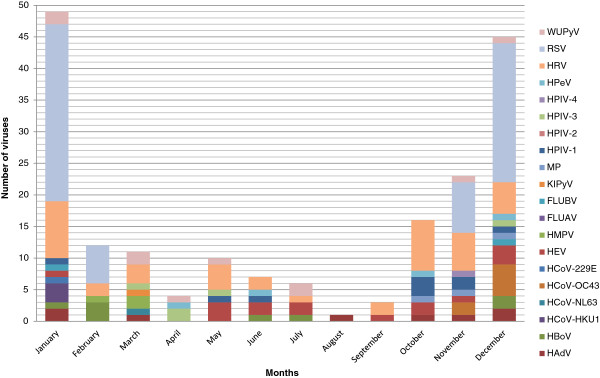
**Seasonality of respiratory pathogens.** Abbreviations: HAdV: human adenovirus, HBoV: human bocavirus, HCoV: human coronaviruses (HKU1, NL63, OC43 and 229E), HEV: human enterovirus, HMPV: human metapneumovirus, FLUAV: influenza A virus, FLUBV: influenza B virus, KIPyV: KI polyomavirus, MP: *Mycoplasma pneumoniae,* HPIV 1–4: human parainfluenza viruses, HPeV: human parechovirus, HRV: human rhinovirus, RSV: respiratory syncytial virus and WUPyV: WU polyomavirus.

There was variability in seasonal detection of respiratory pathogens (Figure 1). RSV, the most detected virus, was found only between November and February, with a peak in December and January. HRV, the second most detected virus, was present during the entire year with two small peaks, one in January and the other in October/November. HEV and WUPyV were also detected throughout the year. Of the HPIVs, HPIV-1 was the most frequent detected virus and detected throughout the year. HCoVs were detected in 12 patients, with HCoV-OC43, being the most frequent coronavirus, only detected in November and December. HPeV, HMPV, FLUBV, KIPyV and *M*. *pneumoniae* were sporadically detected in this study.

### Co-detection of respiratory pathogens

In total, 184 viruses and 3 *M*. *pneumoniae* were detected. Forty-seven (26.6%) respiratory samples had multiple (> 1) respiratory viruses; 38 (21.5%) samples contained 2 respiratory viruses, 8 (4.5%) samples contained 3 viruses, 1 sample contained 2 viruses and *M*. *pneumoniae*, and 1 respiratory sample contained 4 respiratory viruses. RSV and HRV were found in 27 (57.4%) and 28 (59.6%) of the 47 respiratory samples with multiple respiratory pathogens viruses, respectively and RSV and HRV were 12 (25.5%) times co-detected with each other. HCoV-HKU1, HCoV-229E, FLUBV and KIPyV were always co-detected.

### Ct value

**Figure 2:**
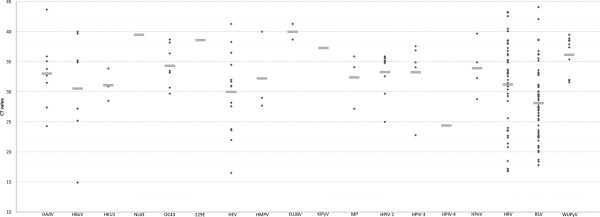
**Mean Ct values of the various respiratory pathogens.** Abbreviations: Ct value: Cycle threshold value, HAdV: human adenovirus, HBoV: human bocavirus, HKU1, NL63, OC43 and 229E: human coronaviruses, HEV: human enterovirus, HMPV: human metapneumovirus, FLUBV: influenza B virus, KIPyV: KI polyomavirus, MP: *Mycoplasma pneumoniae,* HPIV 1–4: human parainfluenza viruses 1–4, HPeV: human parechovirus, HRV: human rhinovirus, RSV: respiratory syncytial virus and WUPyV: WU polyomavirus. Horizontal bars represent group means.

Overall, Ct values between patients in whom only one respiratory pathogen was detected, and patients with > 1 pathogen did not differ significantly (30.5 versus 31.2; p = 0.57). The mean Ct values of the various respiratory pathogens are shown in Figure 2. For RSV the mean Ct value for 36 single virus infections was 28.3 and 27.8 for 28 multiple virus infections (p = 0.74). For HRV the mean Ct value for 15 single virus infections was 30.7 and 31.4 for 27 multiple virus infections (p = 0.76). For HEV the mean Ct value for 6 single virus infections was 33.9 and 27.3 for 9 multiple virus infections (p = 0.057).

### Patients

Patients in whom respiratory pathogens were detected, had a higher rate of hospitalization (116 versus 34; p <0.001) in contrast to patients without respiratory pathogens, as presented in Table 2. Patients in whom respiratory pathogens were detected were also significantly younger (median age 6 months versus 24 months; p <0.001), were more likely to present with feeding problems (p <0.001), dyspnea (p = 0.004), rhinorrhea (p <0.001), wheezing (p = 0.004) and supplemental oxygen requirement (p = 0.009) than those in whom no respiratory pathogen was detected. There was no difference in the duration of hospitalization between the two groups (4.65 versus 3.96 days; p = 0.56).

**Table 2:** Clinical findings of patients with and without respiratory pathogens

	**Number of pathogens detected**		
	**0 (n = 47)**	**≥ 1 (n = 130)**	***p*value**
Median age (months, range)	24 (0–192)	6 (0–153)	0.000
Male (%)	20 (42.6)	78 (60)	0.039
Underlying medical conditions (%)	13 (27.7)	22 (16.9)	0.113
Supplemental oxygen requirement (%)	8 (17.4)	50 (38.5)	0.009
Intensive care unit stay (%)	1 (2.1)	4 (3.1)	0.736
Mean duration of hospitalization (days)	4.0 (range 0–27)	4.7 (range 0–57)	0.565
Prematurity (%)	6 (12.8)	20 (15.4)	0.812
Abnormalities on chest radiograph (%)	11 (23.4)	31 (23.8)	0.951
*Symptoms on presentation* (%)			
General discomfort	28 (59.6)	72 (57.1)	0.773
Feeding problems	12 (26.1)	74 (57.4)	0.000
Cough	27 (58.7)	92 (71.9)	0.099
Fever	24 (51.1)	87 (66.9)	0.054
Dyspnea	13 (27.7)	69 (53.1)	0.003
Vomiting	14 (31.1)	31 (24.2)	0.365
Rhinorrhea	13 (28.9)	77 (61.6)	0.000
Diarrhea	6 (13.3)	20 (15.7)	0.698
Wheezing	8 (17.4)	53 (41.1)	0.004

Patients with one respiratory pathogen detected had a mean hospital stay of 5.45 days in contrast to patients with more than one respiratory pathogen who had a mean hospital stay of 3.03 days (p = 0.024). There was no difference in pre-existent comorbidity, premature delivery and abnormalities on chest radiograph observed between these two groups. In addition, patients with more than 1 respiratory pathogen were more likely to present with the following symptoms when compared to those with 1 respiratory pathogen: vomiting, feeding problems and diarrhea (all p < 0.001). No difference in oxygen supply or admission to an intensive care unit was found.

In a subgroup analysis we compared clinical and microbiological data of patients ≤ 3 months and those older. There were 68 patients ≤ 3 months of age (38.4%) and 109 (61.6%) > 3 months of age. Patients ≤ 3 months of age had a significantly higher rate (98.5 vs. 76.1%; p < 0.001) and duration (7 vs. 3 days; p = 0.001) of hospitalization and more frequently rhinorrhea at presentation (69.1% vs. 42.2%; p < 0.001), than patients > 3 months of age. Patients > 3 months of age presented more often with fever (52.9% vs. 68.8%; p = 0.034) and had a higher level of C-reactive protein (mean 40 mg/l vs. 18 mg/l; p = 0.039) than those ≤ 3 months of age. Four patients ≤ 3 months of age and none of those > 3 months suffered from respiratory insufficiency requiring intubation and mechanical ventilation in an intensive care unit. There was no significant difference in detection rate of respiratory viruses and/or *M*. *pneumoniae* between patients ≤ 3 months and > 3 months of age (79.4% vs. 69.7%; p = 0.15). However, multiple respiratory pathogens were more often detected in patients ≤ 3 months (74.1% vs. 56.6%; p = 0.041).

Compared to other viruses and *M*. *pneumoniae*, the detection of RSV was significantly associated with more abnormalities on chest radiograph (p = 0.015) and with more frequent observation of the following clinical symptoms; fever (p = 0.028), feeding problems, cough, dyspnea, rhinorrhea and wheezing (all p < 0.001). HRV was associated with general discomfort (p = 0.021) and rhinorrhea (p = 0.012) and HEV with fever (p = 0.045), dyspnea (p = 0.007), rhinorrhea (p = 0.014) and wheezing (p = 0.017). HAdV was associated with fever (p = 0.026), HBoV with feeding problems (p = 0.032) and HCoVs with rhinorrhea (p = 0.009).

Patients with respiratory pathogens detected, had more frequently an antibiotic treatment prescribed by the family physician before presentation to the hospital than patients without a positive respiratory (RT)-qPCR (p = 0.048).

Of 139 patients with the clinical diagnosis of RTI, 120 (86.3%) were diagnosed with a viral RTI and 19 (13.7%) with a bacterial RTI. An antibiotic was prescribed to 29 (24.8%) patients with viral infection before the definite diagnosis was made, eventually 17 patients had a positive test result. In 10 (58.8%) of these patients detection of a viral pathogen in the nasopharynx specimen did not result in a switch in antibiotic policy of the pediatrician.

## Discussion

In this retrospective study we evaluated the diagnostic value and the clinical relevance of detecting 19 viruses and *Mycoplasma pneumoniae* in symptomatic pediatric patients attending a pediatric outpatient clinic, emergency department or admitted to the pediatric ward of a large general hospital. At least one respiratory pathogen was detected in 73% of the enrolled patients. These findings are in support of the reports in the literature were viral detection rates of 47-95% are described in children. Possible explanations for the wide differences in detection rates in the literature include heterogeneity in study populations, differences in presenting respiratory symptoms (upper or lower respiratory symptoms), number of respiratory pathogens tested, method used for detection and genetic variability between populations [1,16-18]. RSV and HRV were the most common viruses detected in this patient group, FLUAV and HPIV-2 were not detected during the year. HPIV-2 was possibly not detected due to a biennial cycle of HPIV-2. A plausible explanation for the lack of detection of FLUAV is the fact that the influenza A(H1N1)pdm09 circulated earlier (October-December 2009) than expected and our study was conducted in 2010 and another explanation for the low incidence is the result of a nationwide successful (> 95% coverage) large influenza vaccination campaign against influenza A(H1N1)pdm09 in 2009 for children aged between 6 months to 4 years.

Overall, in 27% of the patients, multiple respiratory viruses were found, comparable to other studies were the co-infection rate ranged from 17% to 41% [17,19]. As expected, the majority of the respiratory pathogens were detected in the younger patients. The higher detection rate of respiratory pathogens among younger children has been ascribed to a higher infection rate, lower viral clearance rate due to a still developing specific immune system against these viruses and higher infection pressure associated with day care attendance and living conditions such as crowding [1,2,20-22]. Furthermore, the parents of younger children may seek healthcare earlier in the course of disease due to parental anxiety.

The recent introduction of more sensitive molecular methods in the clinical practice has increased the virus detection rates significantly compared to viral culture methods. This has, however, made the interpretation of positive test results more difficult, especially when multiple viruses are found with a low Ct value. Nevertheless, the detection of a virus by RT-qPCR does not necessarily means that it causes symptoms. The presence of a virus, particularly HRV, has to be interpreted carefully as viral RNA could be detected in nasal mucus for up to 5–6 weeks after infection and respiratory viruses are also detected in asymptomatic children [23,24]. For newer viruses, such as WUPyV and KIPyV, causality of respiratory disease remains unclear. Until their role in respiratory disease is known inclusion in a respiratory panel for diagnosis may not be warranted.

In literature conflicting results are reported regarding multiple viral co-infections and its association with the severity of acute disease. In this study, 66.6% of the HRV positive patients HRV was co-detected with another virus compared to 42% of the RSV cases. In addition, most samples with more than 1 virus, HRV and RSV were detected together, in 12 (25.5%) of the 47 samples, respectively. Similar to our data, Aberle et al. [17] found HRV and RSV together in 35.9% of the dual infections and Advani et al. [24] found that in 73% of the co-infections HRV was involved. On the other hand, Greer et al. [25] found HRV in a much lower co-detection rate of 23.6%. Some studies have demonstrated that multiple viral pathogens cause more severe disease [1,17], whereas others did not find any difference in disease severity between the detection of a single and multiple respiratory viruses in respiratory samples, not even with the combination of RSV and HRV [16,19,26]. The present findings support the latter conclusion. We found that patients with multiple respiratory viruses even had a shorter duration of hospitalization and were more likely to have non-respiratory symptoms as diarrhea, vomiting and feeding problems. This is similar to the observation of a shorter duration of hospitalization in patients with RSV/HRV dual infections compared to those with a single infection with RSV by others [19,27]. This suggests that HRV may have a potential protective effect on disease severity. This may be explained by the possible protection of the induced immune response trigged by viral infection from infection with a second virus as postulated by Greer et al. [25] where HRV could protect its host from infection by other viruses.

Detection of a virus in patients with respiratory symptoms would be expected to aid a clinician to refrain from the prescription and continuation of antibiotics. Unfortunately, as shown by the present study this is not the real life clinical practice as only in 40% the antibiotic treatment was ceased. The present findings confirm previous reports that have shown that detection of a viral respiratory pathogen in a large part of the pediatric patients did not influence a change in antibiotic management of individual patients [15,28]. This may be attributed to the young age of the children, severity of clinical symptoms and the general attitude of clinicians to complete antibiotic treatment in children due to concern about bacterial co-infections and development of antibiotic resistance. Identification of viral pathogens can be of importance for both individual patient management and hospital infection control policy. Rapid diagnosis could contribute to a significant reduction in unnecessary laboratory tests and costly imaging, especially in young children, thereby decreasing diagnostic costs. Bonner et al. [29] demonstrated this together with a decreased length of time to discharge in a study were the impact of a rapid influenza test result was determined on patient management. Rapid and sensitive diagnosis of viral infections is also thought to be important to reduce nosocomial transmission as all patients infected with a respiratory virus need to be isolated (contact or droplet precautions). All these data suggest that routine molecular testing for respiratory viral pathogens could be useful to decrease the duration of hospitalization and reduce nosocomial infections.

In addition, in specific patient populations, e.g., after stem cell transplantation or with immunosuppression, identification of the viral pathogen can be important in predicting the clinical course, preventing graft-versus-host disease and graft rejection and, even lifesaving [30,31].

## Conclusion

In conclusion, routine testing of common respiratory pathogens in children with ARTIs may lead to a better understanding of the role of viral pathogens in ARTIs and eventually to improvement of individual patient management. However, prospective studies are required to study the need of routinely using such tests in a general pediatric clinic.

## Competing interests

The authors declare that they have no competing interests.

## Authors’ contributions

JR and CO conceived this project and participated in the study design. EH carried out the experiments. EH and RB collected patients’ data and wrote the manuscript. JR, CO and AB revised the manuscript. All authors read and approved the final manuscript.
